# Inhibitory Effects of Protopanaxadiol-Producing Transgenic Rice Seed Extracts on RANKL-Induced Osteoclast Differentiation

**DOI:** 10.3390/life12111886

**Published:** 2022-11-14

**Authors:** Yongjin Lee, Vipada Kantayos, Jin-Suk Kim, Eui-Shik Rha, Young-Jin Son, So-Hyeon Baek

**Affiliations:** 1 Department of Pharmacy, Sunchon National University, Suncheon 57922, Republic of Korea; 2 Department of Agricultural Life Science, Sunchon National University, Suncheon 57922, Republic of Korea

**Keywords:** protopanaxadiol, RANKL, osteoclast differentiation, NFATc1

## Abstract

(1) Background: Osteoporosis is a disease in which bones are weakened and fractured easily because of various factors. It is mainly observed in elderly and postmenopausal women, and it continues to carry high economic costs in aging societies. Normal bone maintains a healthy state through a balanced process of osteoclast suppression and osteoblast activation; (2) Methods: In this study, osteoclast inhibition was induced by inhibiting osteoclast differentiation using ginseng protopanaxadiol-enriched rice (PPD-rice) seed extract. To analyze the effect of PPD-rice extract on the inhibition of osteoclast differentiation, bone marrow macrophages extracted from mice were treated with PPD-rice and Dongjin seed (non-transformed rice) extracts and analyzed for the inhibition of osteoclast differentiation; (3) Results: The results illustrated that PPD-rice extract reduced the transcription and translation of NFATc1, a modulator of osteoclast formation, decreased the mRNA expression of various osteoclast differentiation marker genes, and reduced osteoclast activity. Moreover, the bone resorptive activity of osteoclasts was diminished by PPD-rice extract on Osteo Assay plates; (4) Conclusions: Based on these results, PPD-rice extract is a useful candidate therapeutic agent for suppressing osteoclasts, an important component of osteoporosis, and it could be used as an ingredient in health supplements.

## 1. Introduction

Increasing societal aging has become a significant concern globally because of its critical impact on economic growth, society, and the prevalence of chronic disease [1]. Osteoporosis is a common disease in elderly and postmenopausal women that increases the risk of fractures by reducing bone mineral content and destroying bone microstructures [2]. Osteoporosis is caused by an imbalance between bone formation and bone resorption because of various causes, and it occurs when bone resorption exceeds bone formation. When osteoporosis occurs, the calcium content of bone tissue decreases, and bone marrow space increases; thus, even a slight impact can result in fractures [3,4]. Bone modeling and remodeling have important roles in bone development, growth, and metabolism. Bone remodeling is a dynamic process in which bone that has already grown is maintained in a healthy state through bone resorption and new bone formation. This process is the result of balanced interactions between osteoblasts and osteoclasts. When the balance is disrupted, bone homeostasis is interrupted, and conditions can progress to osteoporosis or osteopetrosis, resulting in various related diseases [5].

An increase in osteoclastogenesis can result in osteoporosis. The processes related to osteoclast differentiation, activation, and death have been previously identified, and receptor activator of nuclear factor κB ligand (RANKL), a cytokine produced by and secreted from osteoblasts (or activated immune cells), has a decisive role in promoting the differentiation and activity of osteoclasts by binding to receptors on osteoclasts [6,7]. In the mechanism by which myeloid progenitor cells differentiate into osteoclasts, the interaction between receptor activator of nuclear factor κB (RANK), an essential substance induced by various factors, and RANKL is essential. The predominant signaling pathway is RANK-RANKL, and after intracombination, intracellular signaling substances are transferred to NFATc1, a major protein of osteoclast differentiation, through the mitogen-activated protein kinase pathway, among other pathways, to increase the expression of NFATc1. Cathepsin K (CTSK), tartrate-resistant acid phosphatase (TRAP), dendritic cell-specific transmembrane protein (DC-STAMP), osteoclast-associated receptor (OSCAR), and others are involved in cell differentiation, and their increased expression can result in the differentiation of osteoclasts into mature osteoclasts, potentially exacerbating osteoporosis [8,9,10,11,12].

Protopanaxadiol (PPD) is an organic compound that characterizes ginsenoside groups. It is a dammarane-type tetracyclic terpene sapogenin found in ginseng [13,14]. The effects of PPD metabolites inside the human body are unclear. However, in vitro studies, PPD shows strong effects on cytotoxicity of cancer cells and anticancer activity [15,16,17]. In addition, PPD has been reported to alleviate obesity by reducing food intake and body weight in mice [18].

Ginsenoside Rb2 is one member of PPD group, has been reported to inhibit osteoclast differentiation through nuclear factor κB and signal transducer and activator of transcription protein 3 signaling pathways [19]. Researchers illustrated that ginsenoside Rb2, the 20(S)-PPD–type ginsenoside from ginseng, inhibits osteoclast differentiation, indicating that it is a potent treatment for osteoporosis [20,21]. 

In a previous study, we created PPD-rice via genetic modification by transferring the PPD synthase genes (PgDDS and CYP716A47) into the Japonica rice cultivar “Dongjin” [22]. This was an attempt to shorten the complex deglycosylation process of ginsenoside active compounds. PPD-rice can be used to reduce the production complexity and cost of ginseng sapogenin, thereby increasing the agricultural product value of the plant. In this study, the effects of a methanolic extract of PPD-rice on osteoclast inhibition were examined, and based on the results, PPD-rice extract could be a useful material in the treatment of the inhibition of osteoclasts that are responsible for one axis of osteoporosis. 

## 2. Results

### 2.1. Determination of PPD via LC-MS/MS

**Figure 1:**
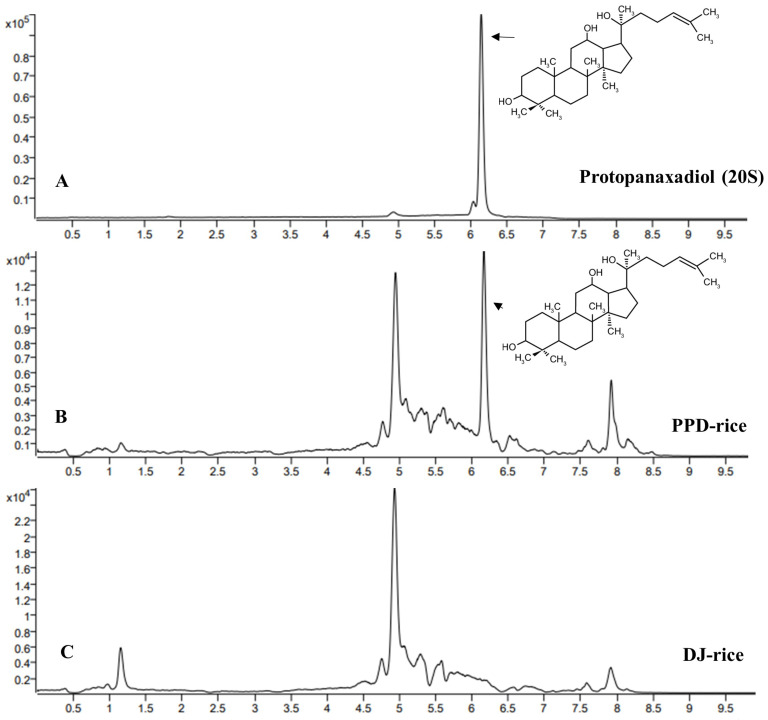
Liquid chromatography-tandem mass spectrometry chromatogram of the methanolic extract of protopanaxadiol (PPD)-enriched rice (PPD-rice). (**A**) Chromatogram of the synthetic standard PPD and (**B**,**C**) chromatogram of methanolic PPD-rice and DJ-rice grains extract (0.1 g/mL).

The methanolic extract of PPD-rice was analyzed for PPD content using LC-MS/MS. The reference PPD standards was observed at a retention time of 6.18 min (Figure 1A). PPD was detected at a retention time of 6.18 min in PPD-rice (Figure 1B), there was no signal in DJ-rice (Figure 1C). The PPD concentration in PPD-rice grains was approximately 7.746 μg/g. 

### 2.2. Inhibitory Effect of PPD-Rice on RANKL-Induced Osteoclast Differentiation 

**Figure 2:**
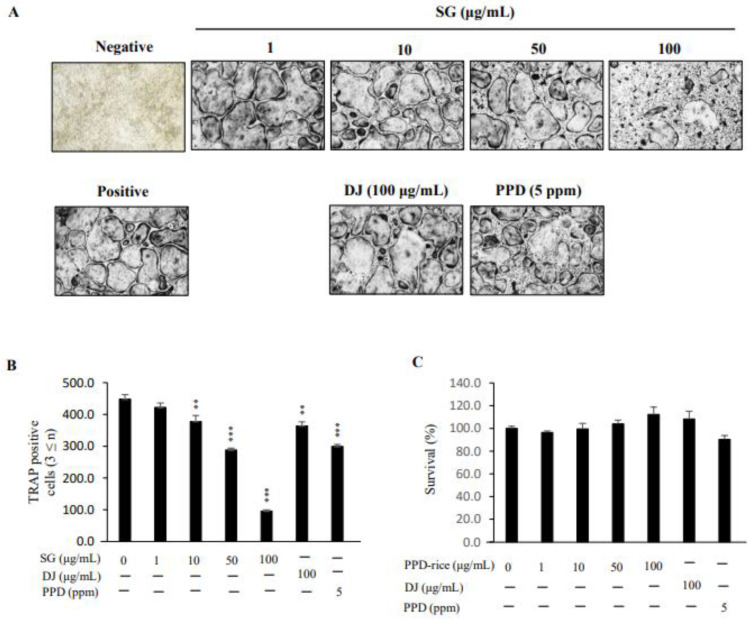
Protopanaxadiol (PPD)-enriched rice (PPD-rice) extract inhibits osteoclast differentiation. (**A**) Osteoclast differentiation was induced for 4 days, and tartrate-resistant acid phosphatase (TRAP) staining was performed. (**B**) TRAP-positive multinucleated cells (three or more nuclei) were considered osteoclasts. (**B**) TRAP-positive multinucleated cells (three or more nuclei) were considered osteoclasts. **, *p* < 0.01; ***, *p* < 0.001 (*n* = 3). (**C**) The effect of PPD-rice extract on bone marrow-derived macrophage viability was evaluated using the CCK-8 assay (*n* = 3). DJ, Dongjin extract.

RANKL exposure induced osteoclasts to differentiate into multinuclear cells, leading to osteoclast differentiation. To investigate the inhibitory effect of PPD-rice on osteoclast differentiation, BMMs treated with M-CSF prior to RANKL treatment were cultured with PPD-rice extract (0, 1, 10, 50, or 100 μg/mL), DJ extract (100 μg/mL), and synthetic PPD (5 ppm). After 4 days of culture, BMMs differentiated into TRAP+ multinuclear cells, and PDD-rice extract more strongly inhibited this differentiation than DJ extract and PPD. The number of TRAP+ cells with three or more nuclei, characteristic of differentiated osteoclasts, was significantly lower in the presence of 100 μg/mL PPD-rice than in the presence of DJ extract or PPD (Figure 2A). DJ extract and PPD inhibited osteoclast differentiation by 19 and 33%, respectively. In the case of PPD-rice extract, the concentration of 10, 50, and 100 μg/mL significantly decreased by 16, 36, and 79% (Figure 2B). PPD-rice extract did not exert cytotoxic effects on BMMs at the concentrations used in this study (1–100 μg/mL) (Figure 2C). Thus, PPD-rice extract inhibited osteoclast differentiation without inducing toxicity. 

### 2.3. Inhibitory Effects of PPD-Rice Extract on RANKL-Mediated Gene Expression

**Figure 3:**
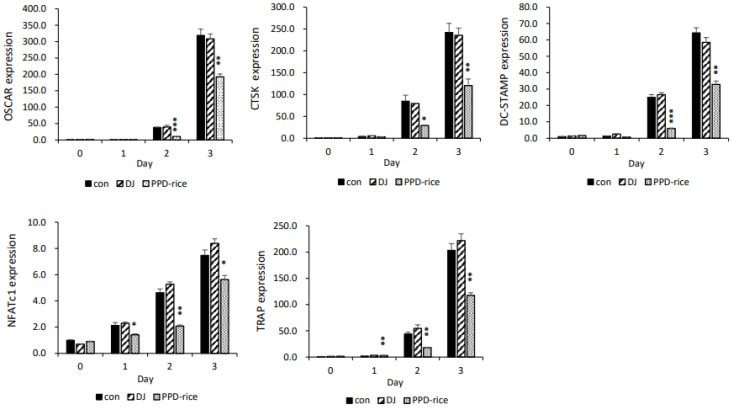
Effects of protopanaxadiol (PPD)-enriched rice (PPD-rice) extract on the receptor activator of nuclear factor κB ligand (RANKL)-mediated mRNA expression of osteoclast-related genes. Effects of PPD-rice extract (100 μg/mL) on RANKL-mediated NFATc1, tartrate-resistant acid phosphatase (TRAP), cathepsin K (CTSK), osteoclast-associated receptor (OSCAR), and dendritic cell-specific transmembrane protein (DC-STAMP) expressions over a 3-day period. Glyceraldehyde-3-phosphate dehydrogenase was used as the internal control. DJ, Dongjin extract (100 μg/mL). *, *p* < 0.05; **, *p* < 0.01; ***, *p* < 0.001.

To investigate the mechanism by which PPD-rice extract inhibited osteoclast differentiation, we investigated its effect on the activation of osteoclast-related genes. The expression of NFATc1, a major transcription factor that regulates osteoclast formation, and marker genes involved in osteoclast differentiation, namely TRAP, DC-STAMP, OSCAR, and CTSK, was analyzed. The mRNA expression of NFATc1 was increased by 3 days of RANKL treatment; however, its expression was significantly suppressed when RANKL-treated cells were treated with PPD-rice extract. Similarly, PPD-rice extract significantly reduced the mRNA expression of TRAP, DC-STAMP, OSCAR, and CTSK (Figure 3). 

### 2.4. PPD-Rice Extract Inhibited RANKL-Induced NFATc1 Protein Expression

**Figure 4:**
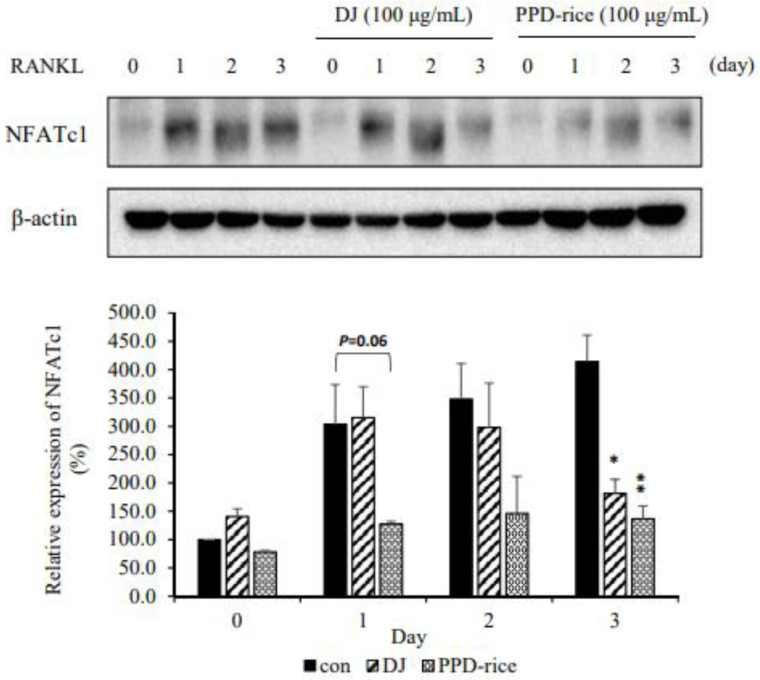
Effect of protopanaxadiol (PPD)-enriched rice (PPD-rice) extract on receptor activator of nuclear factor κB ligand (RANKL)-induced NFATc1 expression. NFATc1 protein expression was investigated under vehicle (0.1% DMSO), Dongjin (DJ) extract, and PPD-rice extract treatment at the indicate times for osteoclast differentiation induced by macrophage colony-stimulating factor and RANKL. Protein expression was analyzed by Western blotting using β-actin as the loading control. *, *p* < 0.05; ** *p,* < 0.01 (*n* = 3).

To examine the effect of PPD-rice extract on NFATc1, Western blotting was performed. NFATc1 expression increased in proportion to the reaction time, and NFATc1 protein expression was significantly decreased by treatment with 100 μg/mL PPD-rice extract (Figure 4), illustrating that the extract inhibits NFATc1 expression and osteoclast formation.

### 2.5. Effects of PPD-Rice Extract on the RANKL-Mediated Bone Resorptive Activity of Osteoclasts

**Figure 5:**
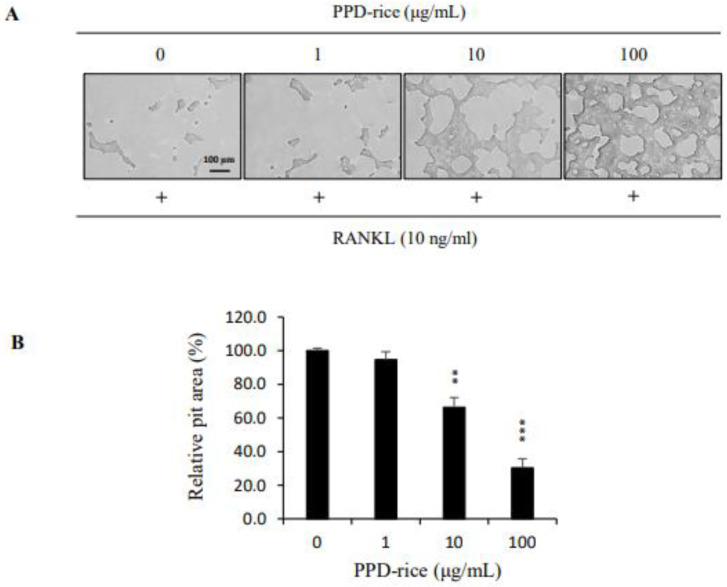
The inhibitory effect of PPD-rice extract on bone resorption by receptor activator of nuclear factor κB ligand (RANKL)-treated osteoclasts. (**A**) The inhibitory effects of PPD-rice expression were measured using Osteo Assay Plates. After 4 days of culture, the pits were observed under a light microscope. (**B**) The effect of PPD-rice extract on the pit area decreased in a concentration-dependent manner. Pit areas were quantified using Image J. **, *p* < 0.01; *** *p,* < 0.001 (*n* = 3).

We investigated the effect of PPD-rice extract on the bone resorption function of mature osteoclasts. Mature osteoclasts created a large pit area because of their bone resorption ability, but PPD-rice extract exposure significantly reduced the pit area in a concentration-dependent manner. PPD-rice extract inhibited osteoclast activity by 5, 34, and 70% at 1, 10, and 100 μg/mL, respectively (Figure 5). 

## 3. Discussion

Osteoporosis is a skeletal disease that increases the risk of fracture because of reductions of bone strength. Bone strength is based on bone mass and bone quality. Bone mass is mainly expressed as bone mineral density, whereas bone quality is reflected by bone structure, the bone replacement rate, mineralization, and micro-damage accumulation. To reduce the risk of bone fracture, it is crucial to both increase bone density and improve bone quality [23,24,25,26,27].

Ginsenoside Rb1 enhances the ability to form bones by increasing alkaline phosphatase activity, mineralization, and the expression of several genes involved in osteoblast differentiation in a concentration-dependent manner [28], but some papers reported that Rb1 did not exhibit any effects in the OVX mouse model [29]. As ginsenoside Rb2 has been reported to promote bone formation and affect bone resorption [30] it is difficult to obtain an active form because of the complexity of its synthesis. Thus, PPD-rice may represent a helpful shortcut to overcome the complicated process of ginsenoside production. PPD production in rice grain is reportedly 3-fold higher than that of its precursor dammarenediol [22]. In this study, the concentration of PPD in rice grain (7.746 μg/g) proved effective for suppressing osteoclast differentiation. We investigated the effect of methanol extracts of PPD-rice and DJ on RANKL-induced osteoclast formation. PPD-rice extract significantly reduced RANKL-induced osteoclast differentiation compared to the effects of DJ extract, although the latter extract also suppressed osteoclast differentiation. Therefore, the mRNA and protein expression of NFATc1 was examined using real-time PCR and Western blotting, respectively, to further clarify the effect of PPD-rice on osteoclast differentiation. PPD-rice extract significantly inhibited RANKL-induced NFATc1 mRNA and protein expression during osteoclast differentiation. In addition, the mRNA expression of TRAP, DC-STAMP, OSCAR, and CTSK was significantly reduced by PPD-rice extract treatment. TRAP is an important osteoclast enzyme and has been used for many years as a marker of bone resorption [31]. OSCAR is an immunological mediator and regulator of osteoclast differentiation [32]. The most characteristic feature of osteoclasts is multinucleation resulting from cell–cell fusion of mononuclear osteoclasts. DC-STAMP is an essential cell–cell fusion regulator [33]. CTSK is responsible for the degradation of type I collagen in osteoclast-mediated bone resorption [34]. The pit resorption assay is used to study osteoclast-mediated bone resorption, and PPD-rice extract inhibited RANKL-induced osteoclast differentiation [35,36,37,38]. A resorptive pit assay was performed to investigate the effect of PPD-rice extract on osteoclast activity. After differentiation of osteoclasts, the activity of bone resorption is confirmed by applying it to the bone disc. Since the osteoclasts used in this study are primary cells directly extracted from the femur and tidia of a mouse, the bone resorption activity of these osteoclasts was investigated, and it was found that the PPD-rice extract inhibited the osteoclast activity of bone marrow.

In this study, PPD-rice extracted prevented osteoclast differentiation by inhibiting osteoclast formation-related factors by suppressing the expression of NFATc1 through RANKL–RANK binding. PPD-rice extract had greater inhibitory efficacy than DJ extract and synthetic PPD. Since DJ extract and PPD-rice extract have a synergistic effect in suppressing osteoclasts, a major factor in osteoporosis, PPD-rice extract is an important candidate as a material for health functional foods and therapeutics. However, there are some limitations that people are biased towards research due to GMO issues. As GMOs, soybeans and corn are widely consumed around the world, and they are also applied to fruits and fish. However, since the commercialization of GMOs, concerns about safety have continued. In the future, the safety and superiority of PPD-rice extract will be verified through animal studies.

## 4. Materials and Methods

### 4.1. Plant Material Preparation for Liquid Chromatography-Tandem Mass Spectrometry (LC-MS/MS)

Ten grams of PPD-rice (transgenic rice of the parental DJ rice) and DJ rice (wild-type) grains powder were soaked in 100 mL of methanol and extracted using an ultrasonic-assisted extraction method for 1 h. After high-speed centrifugation, the solvent was removed from the supernatant using a rotary evaporator at 40 °C. Then, the excess water was removed using the freeze-drying method. The crude extract was filtered through a 0.45-μm filter before injection. All samples were analyzed using an LC-MS/MS system (Agilent 6460 Triple Quadrupole). The PPD components were separated using a column oven (3.0 × 50 mm, 2.7 microns, Agilent Poroshell 120 EC-C18) at 40 °C. The mobile phases consisted of distilled water (solvent A) and acetonitrile (solvent B), and the flow rate was 0.3 mL/min. The injection volume was set at 5 µL. Gradient elution was programmed as follows: 25% solvent B (0–5 min), 5% solvent B (6–8 min), and 25% solvent B (8.10–10 min). The mass spectrometer was connected to an electrospray ionization source and interfaced in the positive ion mode. The nebulizing gas flow rate was 5 L/min.

### 4.2. Preparation and Differentiation of Osteoclasts

We conducted in vitro experiments using a previously reported method [12]. Cell cultures were passaged every 3 days at 37 °C in 5% CO_2_. The experimental protocol was approved by the Sunchon National University Animal Education Institution Use Committee (SCNUIACUC; permission number SCNU IACUC 2021-06).

Bone marrow-derived macrophages (BMMs) were collected from the femurs and tibiae of 5-week-old male ICR mice (*n* = 2; Raon Bio, Yongin, Korea). BMMs were seeded into a 96-well plate at 1 × 10^4^ cells/well with macrophage colony-stimulating factor (M-CSF) (30 ng/mL; PEPROTECH, Cranbury, NJ, USA) in α-MEM supplemented with 10% FBS, and the next day, cells were treated with RANKL (10 ng/mL; R & D Systems, Minneapolis, MN, USA) and PPD-rice extract for 4 days.

### 4.3. TRAP Staining for Identification of Osteoclast Differentiation

The cells were washed with phosphate-buffered saline and fixed with 3.7% formalin for 5 min. The fixed cells were reacted with Triton X-100 (0.1%) for 10 min and then treated with TRAP solution (Sigma–Aldrich, St. Louis, MO, USA) for 10 min in the dark at room temperature. TRAP-positive multinucleated cells (nuclei ≥ 3) were counted as mature osteoclasts.

### 4.4. Cytotoxicity Assay of PPD-Rice Extract

To investigate the effect of PPD-rice extract on cell viability, BMMs were cultured for 3 days in the presence of PPD-rice extract. BMMs were seeded at 1 × 10^4^ cells/well and cultured in 10% FBS-supplemented α-MEM containing M-CSF (30 ng/mL). Cell viability was evaluated using the Cell Counting Kit-8 assay (DOJINDO Molecular Technologies Inc., Kumamoto, Japan) according to the manufacturer’s protocol.

### 4.5. Real-Time PCR

Real-time PCR was performed as previously described [39]. BMMs were incubated with M-CSF (30 ng/mL) in 10% FBS-supplemented α-MEM and activated with RANKL (10 ng/mL) for 0, 1, 2, or 3 days in the presence of PPD-rice and Dongjin (DJ) extracts. PCR primer sets (Table 1) were designed using an online primer3 program [40]. Total RNA was isolated using TRIzol reagent (Thermo Fisher Scientific Inc., Waltham, MA, USA), and cDNA was synthesized using an M-MLV cDNA synthesis kit (Enzynomics, Daejeon, Republic of Korea). Real-time qPCR was performed using TOPreal qPCR 2× PreMIX (Bio-Rad, Hercules, CA, USA) in a real-time PCR detection system (Bio-Rad). The mRNA levels of the genes were determined using the 2^−ΔΔCt^ method. Glyceraldehyde-3-phosphate dehydrogenase was used as the internal standard [41].

### 4.6. Western Blotting

Western blotting was conducted as previously described [39]. Briefly, BMMs were incubated with RANKL (10 ng/mL) and M-CSF (30 ng/mL) in 10% FBS-supplemented α-MEM for 0, 1, 2, or 3 days in the presence of vehicle (DMSO), DJ extract, and PPD-rice extract. Harvested cells were lysed in lysis buffer containing a protease inhibitor, and protein content was quantified using the Bradford assay. Isolated proteins were separated on 10% SDS-PAGE gels and transferred to a PVDF membrane (Millipore, Burlington, MA, USA). The membrane was incubated with primary antibody (anti-NFATc1) at 4 °C overnight. The β–actin was used as the internal control.

### 4.7. Bone Pit Formation Assay

An experiment using an Osteo Assay Plate (24 well plate; Corning Inc., Corning, NY, USA) was performed to measure the bone resorptive activity of BMMs. After incubating BMMs for 3 days in 10% FBS-supplemented α-MEM containing M-CSF and RANKL to induce differentiation, cells were treated with vehicle or PPD-rice extract. The bone pit areas were observed under a light microscope (magnification, ×50; Leica Microsystems, Wetzlar, Germany) and measured using Image J software (NIH, Bethesda, MD, USA).

### 4.8. Statistical Analysis

All quantitative data generated in this study were expressed as the mean ± standard deviation of three replicate experiments. Statistical differences were identified using Student’s *t*-test. *p* < 0.05 denoted statistical significance.

## 5. Conclusions

PPD-rice extract inhibited osteoclastogenesis by decreasing the expression of NFATc1 during RANKL-induced osteoclast differentiation. The extract also decreased the expression of other factors involved in osteoclast differentiation (TRAP, DC-STAMP, OS-CAR, and CTSK). In addition, PPD-rice extract decreased resorption activity. Therefore, PPD-rice can be applied as a useful functional food or therapeutic agent for the inhibition of osteoclasts that play an important role in inducing osteoporosis.

## Figures and Tables

**Table 1:** Sequences of primers used in this study.

Gene of Interest	Primer Sequence (5′→3′)	
	Sense	Anti-Sense
NFATc1	GGGTCAGTGTGACCGAAGAT	GGAAGTCAGAAGTGGGTGGA
CTSK	GGCCAACTCAAGAAGAAAAC	GTGCTTGCTTCCCTTCTGG
DC-STAMP	CCAAGGAGTCGTCCATGATT	GGCTGCTTTGATCGTTTCTC
OSCAR	CTGCTGGTAACGGATCAGCTC	CCAAGGAGCCAGAACCTT
TRAP	GATGACTTTGCCAGTCAGCA	ACATAGCCCACACCGTTCTC
GAPDH	AACTTTGGCATTGTGGAAGG	ACACATTGGGGGTAGGAACA

CTSK, cathepsin K; DC-STAMP, dendritic cell-specific transmembrane protein; OSCAR, osteoclast-associated receptor; TRAP, tartrate-resistant acid phosphatase; GAPDH, glyceraldehyde-3-phosphate dehydrogenase

## Data Availability

Not applicable.
